# Inheritance of perturbed methylation and metabolism caused by uterine malnutrition via oocytes

**DOI:** 10.1186/s12915-023-01545-x

**Published:** 2023-02-24

**Authors:** Shou-Bin Tang, Ting-Ting Zhang, Shen Yin, Wei Shen, Shi-Ming Luo, Yong Zhao, Cui-Lian Zhang, Francesca Gioia Klinger, Qing-Yuan Sun, Zhao-Jia Ge

**Affiliations:** 1 College of Life Sciences, Institute of Reproductive Sciences, Key Laboratory of Animal Reproduction and Germplasm Enhancement in Universities of Shandong, Qingdao Agricultural University, Qingdao, 266109 People’s Republic of China; 2 Reproductive Medicine Center, People’s Hospital of Zhengzhou University, Henan Provincial People’s Hospital, Zhengzhou, 450003 People’s Republic of China; 3 Fertility Preservation Lab and Guangdong-Hong Kong Metabolism & Reproduction Joint Laboratory, Reproductive Medicine Center, Guangdong Second Provincial General Hospital, Guangzhou, 510317 People’s Republic of China; 4 State Key Laboratory of Animal Nutrition, Institute of Animal Sciences, Chinese Academy of Agricultural Sciences, Beijing, People’s Republic of China; 5 Histology and Embryology, Saint Camillus International University of Health Sciences, Rome, Italy

**Keywords:** Oocytes, methylation, Undernourishment in utero, Metabolic disorders, Transgenerational transmission

## Abstract

**Background:**

Undernourishment in utero has deleterious effects on the metabolism of offspring, but the mechanism of the transgenerational transmission of metabolic disorders is not well known. In the present study, we found that undernourishment in utero resulted in metabolic disorders of female F1 and F2 in mouse model.

**Results:**

Undernutrition in utero induced metabolic disorders of F1 females, which was transmitted to F2 females. The global methylation in oocytes of F1 exposed to undernutrition in utero was decreased compared with the control. KEGG analysis showed that genes with differential methylation regions (DMRs) in promoters were significantly enriched in metabolic pathways. The altered methylation of some DMRs in F1 oocytes located at the promoters of metabolic-related genes were partially observed in F2 tissues, and the expressions of these genes were also changed. Meanwhile, the abnormal DNA methylation of the validated DMRs in F1 oocytes was also observed in F2 oocytes.

**Conclusions:**

These results indicate that DNA methylation may mediate the transgenerational inheritance of metabolic disorders induced by undernourishment in utero via female germline.

**Supplementary Information:**

The online version contains supplementary material available at 10.1186/s12915-023-01545-x.

## Background

With the rapid development of social-economy, the incidence of non-communicable diseases such as diabetes, obesity, and cardiovascular diseases is rising quickly. Epigenetic modifications which are sensitive to environmental changes are regarded to play a key role in the origination of diseases, which is called non-Mendelian phenotypic inheritance [1]. Recent studies have demonstrated that early life environment plays a key role in the adult non-communicable diseases, which may be mediated by epigenetics [2]. For example, paternal diet defines the transgenerational abnormality of metabolic gene expression [3] and intergenerational obesity [4]. We recently showed that paternal prediabetes caused sperm methylome changes that could be transmitted to the next generation [5]. The inheritance of metabolic diseases not only can be mediated by male germline [6], also possibly by female germline [7]. That is because the effects of environment on males and females are different. The sex dimorphism of effects of environmental conditions on offspring is widely observed. For example, high-fat-diet has a more severe effect on the metabolism of male offspring compared with female offspring [8]. In humans, male offspring are also more severely affected by gestational diabetes mellitus than female offspring [9]. Recently, it is reported that female offspring is more severely affected by malnutrition in utero compared with male offspring [10]. It is demonstrated that DNA methylation changes can be inherited via female germline in *A*^*vy*^ and *Axin* mouse models [11]. In *C. elegans*, the message from high temperature can be inherited to at least 14 generations through oocytes [12]. Therefore, we hypothesize that the inheritance of metabolic disorders induced by malnutrition in utero could be mediated by DNA methylation through oocytes. To achieve our aims, we produced an undernourished mouse model as described by Radford et al. [13] and examined the global methylation status in F1 oocytes and the inheritance of methylation changes and metabolic disorders.

## Results

### Undernourishment in utero induced metabolic disorders in F1 offspring

**Fig. 1:**
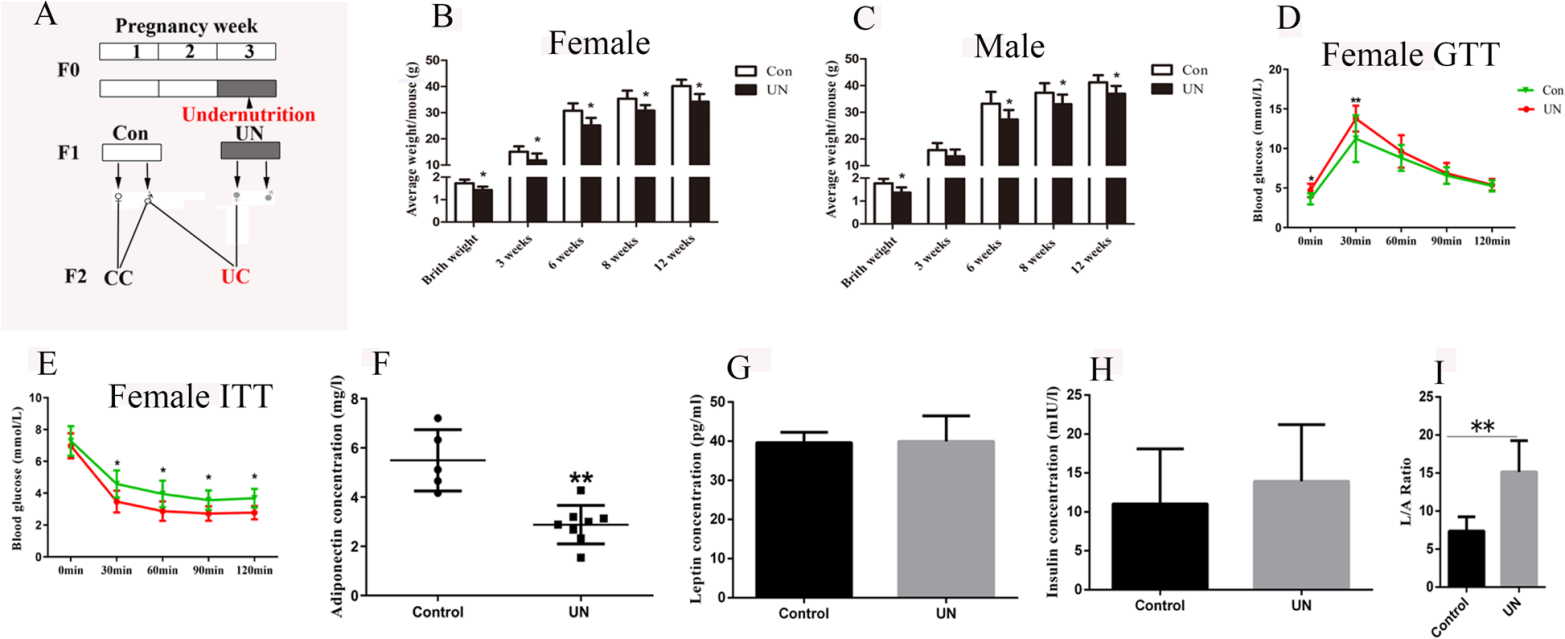
Effects of undernutrition in utero on metabolism of UN female. **A** The schedule of breeding. F1 and F2 were produced as shown. UN, undergoing undernourishment in utero; Con, the control group; CC, female control mated with male control; UC, female UN mated with male control. **B** and **C** The variation of body weight from birth to 12 weeks (*n*_female_ ≥ 17, *n*_male_ ≥ 17) of age. **D** and **E** GTT and ITT were tested at 8 weeks of age for females. **F**–**H** The plasma concentrations of leptin, adiponectin, and insulin were examined, and the statistical difference was tested by Student’s *t* test. **I** The L/A ratio (leptin/adiponectin) was calculated. *p < 0.05; **p < 0.01. Average data are showed as mean ± SD

We established a mouse model as described by previous studies [14] and bred F1 and F2 generations (Fig. 1A) [15]. F1 offspring experiencing undernourishment in utero (UN) were smaller from birth to 12 weeks of age for both males (*n* ≥ 18) and females (*n* ≥ 23, Fig. 1B, C). The blood glucose was higher in the UN group (*n* ≥ 11) than that in the control (*n* ≥ 12) at 8 weeks of age (Additional file 1: Fig. S1A, B). However, it was similar between the control (*n* ≥ 13) and UN groups (*n* ≥ 18) at 12 weeks of age (Additional file 1: Fig. S1C, D). We further tested the glucose (GTT) and insulin tolerance (ITT) and found that both GTT and ITT were significantly altered by undernourishment in utero for female UN offspring (*n* = 10, *p* < 0.05, Student’s *t*-test, Fig. 1D, E). Disturbed GTT and ITT were also observed for male UN offspring (*n* = 10, Additional file 1: Fig. S1E, F) at 8 weeks of age.

Leptin, insulin, and adiponectin are three of the most important hormones regulating energy metabolism and body weight in mammals. Abnormal leptin, adiponectin, and insulin levels are associated with obesity, diabetes, and other metabolic disorders [16, 17]. We investigated the plasma concentrations of adiponectin, insulin, and leptin, and found that the plasma adiponectin concentration was significantly lower in female UN offspring compared with the control (UN 2.811 ± 0.2798 mg/l *n* = 8, control 5.663 ± 0.6312 mg/l *n* = 5, *p* = 0.0006, Student’s *t*-test, Fig. 1F). The plasma concentrations of leptin and insulin in female UN offspring (*n* = 8) were similar to the control (*n* = 9, leptin *p* = 0.9153; insulin *p* = 0.9288, Student’s *t*-test, Fig. 1G, H). The leptin/adiponectin ratio (L/A ratio) is a better indicator of metabolic diseases, such as obesity and diabetes mellitus [18]. The L/A ratio in F1 female UN offspring was about 2-fold of that in the control (*p* = 0.0012, Student’s *t*-test, Fig. 1I). For male UN offspring, the L/A ratio in UN was higher than that in control (Additional file 1: Fig. S1G), but the concentration levels of leptin, adiponectin, and insulin were not significantly affected (*n* = 8, Additional file 1: Fig. S1H, I, and J).

### Intergenerational metabolic disorders induced by undernourishment in utero via female germline

**Fig. 2:**
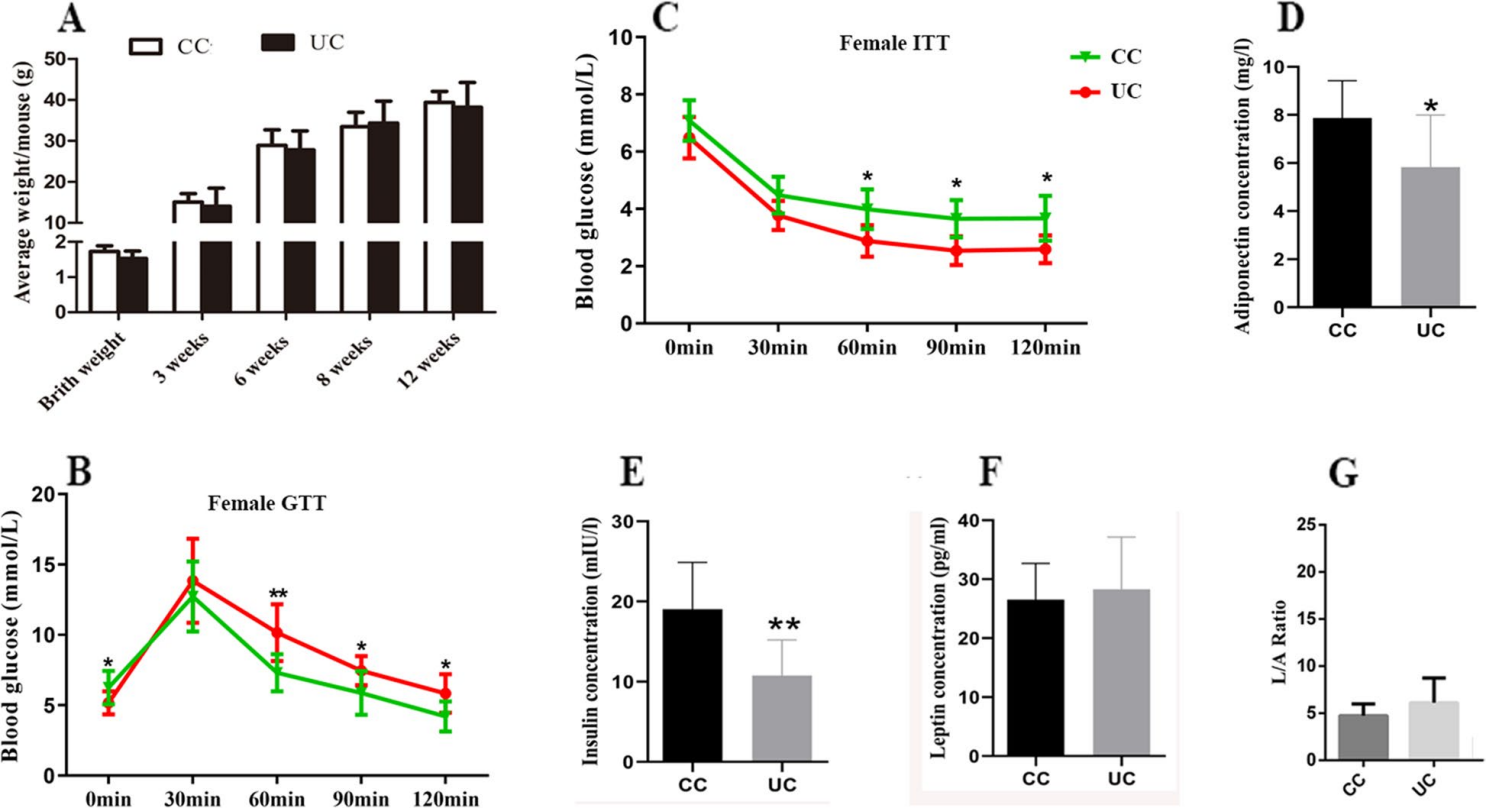
The intergenerational transmission of perturbed metabolism in UC and CU females. **A** The average body weight changes of female UC. **B** and **C** The GTT and ITT changes for female UC, *X*-axial is the time point when glucose level was tested. **D**–**F** The plasma concentrations of leptin, adiponectin, and insulin. **G** The L/A ratio changes of female UC. The statistical difference was tested by Student’s *t* test, and * *p* < 0.05; **p < 0.01. Average data are showed as mean ± SD

To confirm the inheritance of metabolic disorders via female germline, we bred F1 control and UN females with control males; offspring of these were marked as CC (F2 offspring of control females) and UC (F2 offspring of UN females) (Fig. 2A). The body weight of UC females (*n* ≥ 14 from 5 litters) at birth and at 12 weeks of age (Fig. 2A) was similar to CC (*n* ≥ 12 from 5 litters). We further tested ITT and GTT, and they were significantly altered in UC females (*n* = 10 from 5 litters) compared with CC (*n* = 10 from 5 litters, *p* < 0.05, Fig. 2B, C). The plasma concentrations of adiponectin (*p* = 0.0301) and insulin (*p* = 0.0039) in female UC offspring (Student’s *t*-test, *n* = 12 from 6 litters) were significantly decreased compared with CC (*n* = 10 from 6 litters, Fig. 2D, E). The plasma concentration of leptin was similar between CC and UC (Fig. 2F). In addition, the L/A ratio increased 1.43-fold in female UC offspring compared to CC (*p* = 0.1539, Fig. 2G).

For the UC males, the GTT, ITT, and body weight were not affected (*n* ≥ 10 from ≥5 litters, Additional file 1: Fig. S2A, B and C). Although the plasma concentrations of insulin, adiponectin, and leptin were similar between CC (*n* = 5 from 4 litters) and UC males (*n* = 8 from 4 litters, Additional file 1: Fig. S2 D, E, and F), the L/A ratio in UC males was significantly higher than that in CC (Additional file 1: Fig. S2G).

### Global hypo-methylation in UN oocytes of F1 offspring exposed to undernourishment in utero

**Fig. 3:**
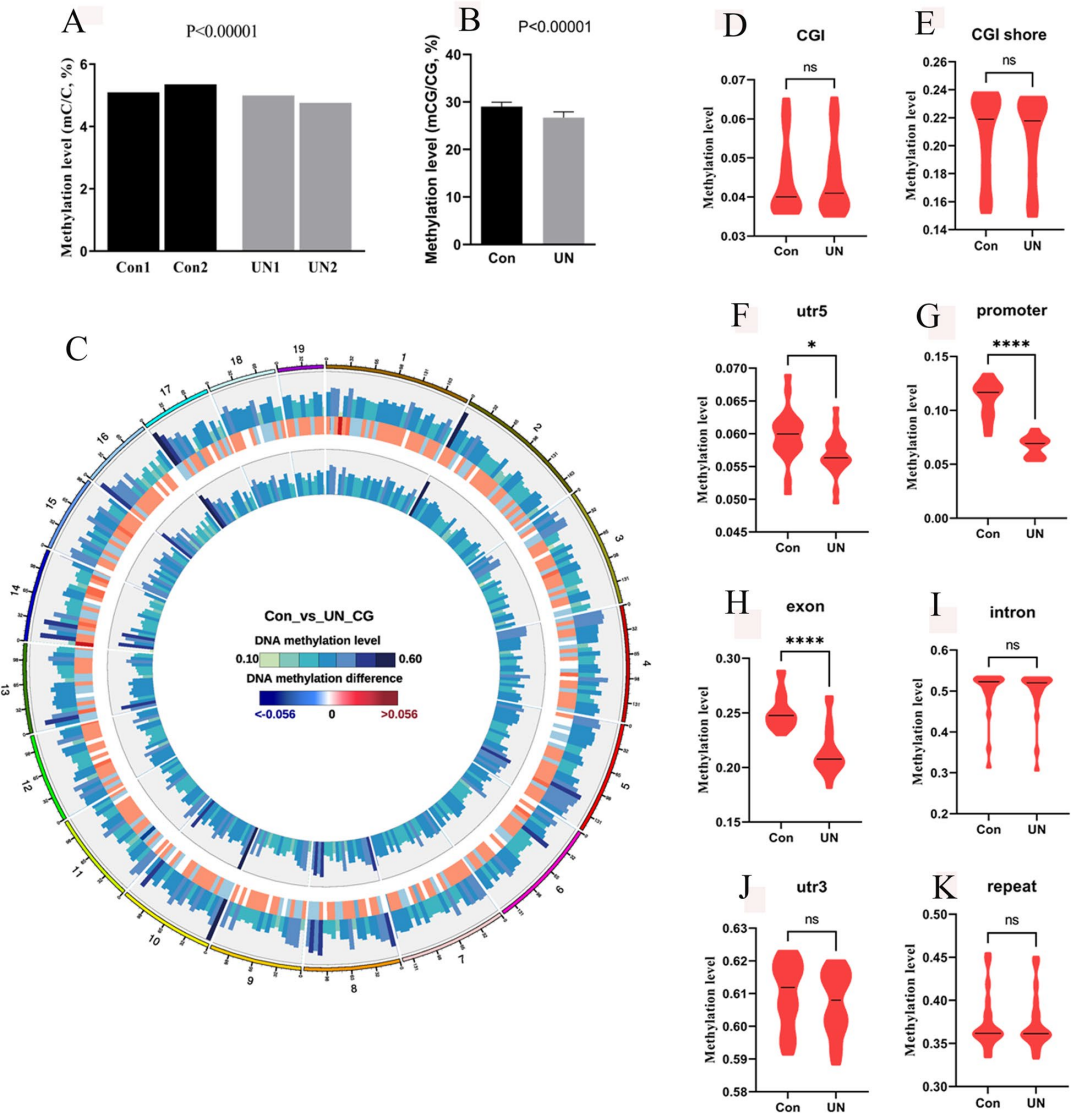
Global hypo-methylation in F1 UN oocytes. **A** Global C hypo-methylation was observed in UN oocytes, two repeats for each group. **B** Global hypo-methylation was also observed for CG sites in UN oocytes. **C** Global methylation level and methylation difference. The circles from outer to inner respectively represent chromosomes, the control methylation level in oocytes, the methylation difference between groups, the methylation level in UN oocytes, and graph legends. **D**–**K** The CG methylation level at different regions. *p < 0.05, ****p < 0.0001. Average data are showed as mean ± SD

Considering the role of DNA methylation in the inheritance of metabolic disorders, we investigated the effects of undernourishment in utero on global methylation in F1 UN adult oocytes. A total of 100 MII oocytes were pooled to make two pools for each group, each pool comprising seven individuals from five independent litters. Base-resolution methylome was obtained by bisulfite sequencing (BS-seq) for single cell. C (cytosine) methylation was evaluated using Bismark at the sequencing depth ≥ 5 and *q*-value ≤ 0.01. Global C methylation (mC) level was lower in UN oocytes than that in control (Fig. 3A). According to the context, mC has three subtypes: mCG, mCHG, and mCHH (H = A, C or T). The proportion of mCG in three types was significantly decreased in UN oocytes compared with the control (*p* < 0.00001, Additional file 1: Fig. S3A).

mCG has a more important role in regulating gene expression and biological function; thus, we further analyzed CG methylation in oocytes. Hypo-methylation of CG was observed in oocytes of F1 generation (Fig. 3B), and this was distributed at all chromosomes (Fig. 3C). Further analysis in genomic features and gene regions showed that significant hypo-methylation was mainly distributed in promoters, exons, UTR5s, upstream 2kb regions of TSS (transcriptional start site), and downstream 2kb regions of TES (transcriptional end site) (Fig. 3D–K, and Additional file 1: Fig. S3B, C). These results suggest that the global methylation in UN oocytes is decreased by undernourishment in utero.

### Effects of undernourishment in utero on the DMRs methylation level in oocytes of F1 generation

**Fig. 4:**
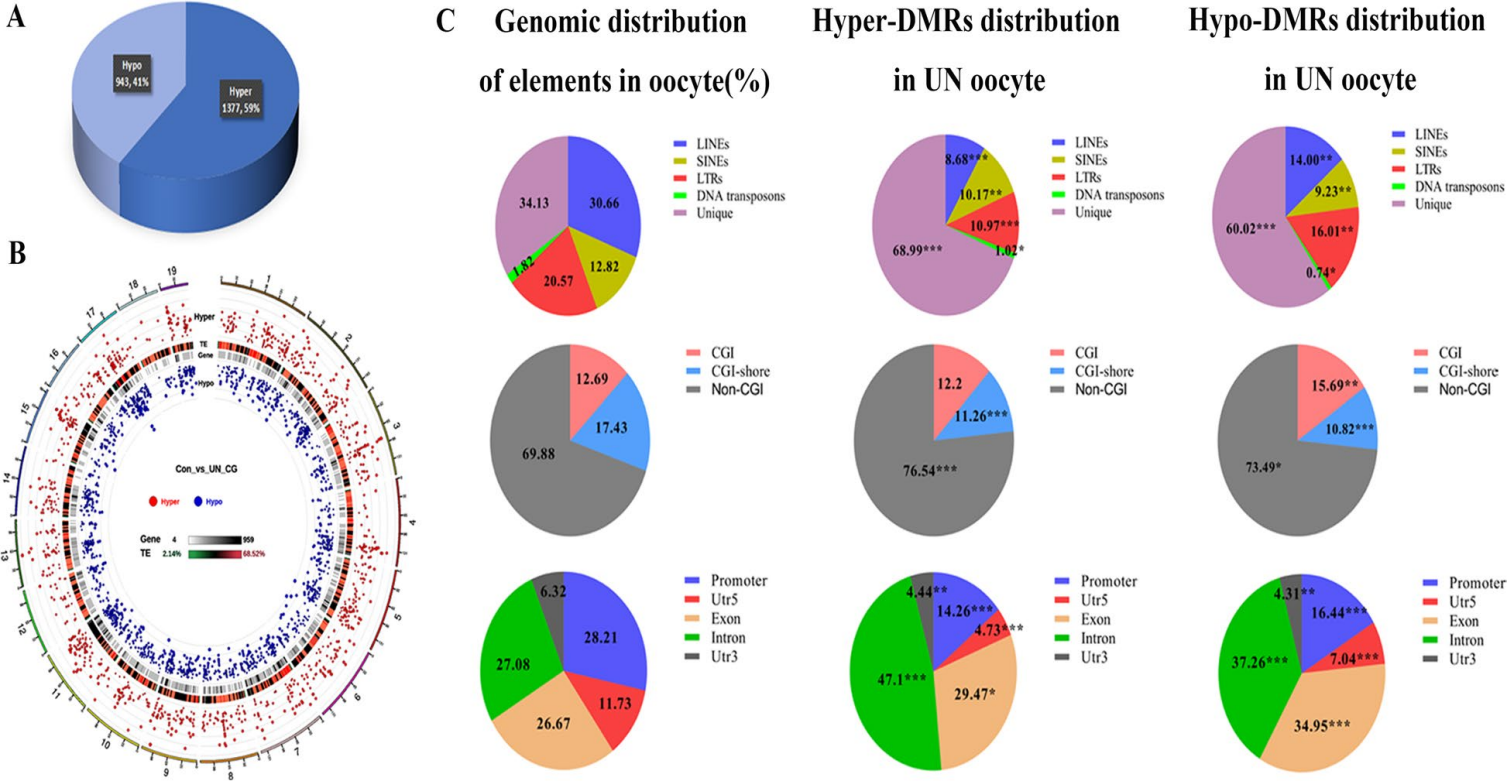
DMRs methylation analysis in F1 UN oocytes. **A** Identified DMRs in UN oocytes including hyper-DMRs and hypo-DMRs. **B** DMRs was distributed at all chromosomes. The circles from outer to inner respectively represent chromosomes, statistical hyper-DMRs value log5 (|area Stat|), heat map of transcriptional elements (TE) and repeats, heat map of genes, statistical hypo-DMRs value log5 (|area Stat|), and graph legend. **C** Distribution of DMRs at different genomic regions, the left panel is the distribution of elements in oocytes; the middle panel is the distribution of hyper-DMRs in different elements, and the right panel is the distribution of hypo-DMRs in different elements. *p < 0.05; **p < 0.01, ***p < 0.001

To better understand the effects of undernourishment in utero on genomic methylation in UN oocytes, we scanned the differentially methylated regions (DMRs) between UN and control oocytes at the number of CG ≥ 4 and the absolute difference of methylation ≥ 0.2. We identified a total of 2320 DMRs, of which 943 were hypo-methylated DMRs (hypo-DMRs, 41%) and 1378 were hyper-methylated DMRs (hyper-DMRs, 59%) in UN oocytes compared with control (Fig. 4A). And DMRs were located at all chromosomes (Fig. 4B). Furthermore, we examined the distribution of DMRs in genomic features including repeats and gene regions. Hyper-DMRs were significantly depleted from repeat regions, CGI-shores, UTR3s, UTR5s, and promoters, but enriched in exons and introns (Fig. 4C). Notably, hypo-DMRs were significantly enriched in CGIs, exons, and introns, but depleted from the other regions (repeat regions, CGI-shores, UTR3s, UTR5s, and promoters, Fig. 4C). These results indicate that DMRs are not randomly distributed throughout the genome in UN oocytes.

DNA methylation patterns in promoters play an essential role in regulating gene expression. We identified 278 genes with DMRs in promoters, including 118 genes with hypo-DMRs and 160 genes with hyper-DMRs (Additional file 1: Table S1). To further understand the roles of DMRs in biological process, we analyzed the KEGG (Kyoto Encyclopedia of Genes and Genomes) enrichment of genes with DMRs in promoters in UN oocytes using KOBAS [19]. These genes were significantly (corrected *p* < 0.05) enriched in 10 KEGG pathways (Additional file 1: Table S2). The KEGG analysis showed that the genes with differentially methylated promoters were significantly enriched in metabolic pathways (22 genes, Table S3): 10 genes with hypo-DMRs, and 12 genes with hyper-DMRs. Some of genes enriched in metabolic pathways were relative to lipid synthesis, glucose metabolism, insulin secretion, mitochondrial function, fatty acid metabolism, insulin resistance, diabetes, and obesity, for instance *Aacs* (acetoacetyl-CoA synthetase), *Acsl6* (Long-chain acyl-CoA synthetase 6), *Adcy8* (Adenylate cyclase), *Bcat2* (branched-chain aminotransferase 2), *Coq5* (Coenzyme Q5), *Got1/2* (aspartate aminotransferase 1/2), *Isyna1* (Inositol-3-Phosphate Synthase 1), *Pde4d* (cAMP phosphodiesterase 4d), *Pde10a* (cAMP phosphodiesterase 10a), *Pde8b* (cAMP phosphodiesterase 8b), *Plpp2* (phospholipid phosphatase 2), *Shmt2* (serine hydroxymethyltransferase 2), *Tkt* (transketolase), *car8* (carbonic anhydrase 8), and *Pigl* (phosphatidylinositol glycan anchor biosynthesis class L). These results indicate that these genes with DMRs in the promoter regions may play a crucial role in the metabolic disorders of F2 females.

### Methylation level of the DMRs located at the promoters of metabolic-related genes in UN oocytes

**Fig. 5:**
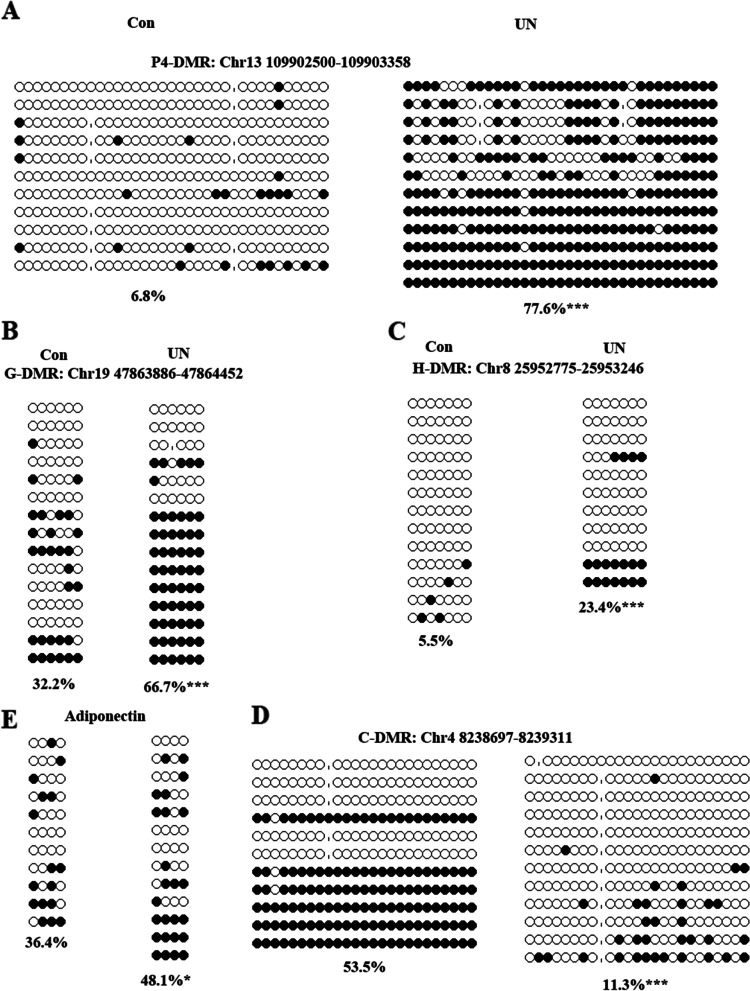
Confirmation of methylation changes of selected DMRs in F1 UN oocytes. **A** P4-DMR. **B** G-DMR. **C** H-DMR. **D** C-DMR. **E** Adiponectin. *p < 0.05; ***p < 0.001. Black circle: methylated CG site; white circle: unmethylated CG site

To further confirm the methylation of the DMRs located at the promoters of metabolic-related genes in UN oocytes, we tested the methylation levels of some DMRs using BS: 4 hyper-DMRs located at the promoters of *Hgsnat* (H-DMR), *Gsto2* (G-DMR), *Car8* (C-DMR), *Pde4d* (P4-DMR), and 3 hypo-DMRs located at the promoters of *Gnas* (Gn-DMR), *Pde8b* (P8-DMR), and *Acsl6* (A-DMR). A total of 80-120 oocytes were used for each DMR analysis. For hyper-DMRs, the methylation levels of H-DMR, G-DMR, and P4-DMR in UN oocytes were significantly higher than that in the control (Fig. 5A, B, and C). But the methylation level of C-DMR was significantly lower in UN oocytes compared to the control (Fig. 5D). For hypo-DMRs, the methylation levels of A-DMR and Gn-DMR were significantly lower in UN oocytes (Additional file 1: Fig. S4A, B). The methylation levels of P8-DMR were similar between UN and control oocytes (Additional file 1: Fig. S4C). These indicate that some regions were false-positives.

Martinez et al. found that in utero malnutrition altered the methylation level of 5′ UTR region of *Lxra* in F1 sperm which may play a key role in the metabolic disorders of F2 [20]. Therefore, we further examined the methylation level of *Lxra*, but it was not significantly affected in UN oocytes (Additional file 1: Fig. S4D).

*Adiponectin* and *Leptin* are essential for the onset of metabolic disorders, such as diabetes and obesity, and their expressions are regulated by the methylation level in promoters. The global methylation sequencing results showed that the methylation levels in promoters of *Leptin* and *Adiponectin* were higher in UN oocytes compared with the control (Additional file 1: Fig. S5A and B). But just 3 CpG sites were covered for *Adiponectin* and 4 CpG sites were covered for *Leptin* in the genomic sequencing results. To more accurately assess the methylation of *Leptin* and *Adiponectin* in UN oocytes, we validated it by BS. We found that DNA methylation level in the promoter of *Leptin* in UN oocytes was similar to that of the control (Fig. Additional file 1: S4E). But the DNA methylation level in the promoter of *Adiponectin* was significantly higher in UN oocytes compared with the control (Fig. 5E).

It is known that the methylation pattern in oocytes is different from somatic cells. To exclude somatic cell contamination from our result, we examined the DNA methylation status of paternally imprinted gene *H19* and maternally imprinted gene *Igf2r* in oocytes using COBRA. All the samples for *H19* were undigested by both of *Taqα I* and *Rsa I*, and the samples for *Igf2r* were digested by both of *Taqα I* and *Bstu I* (Additional file 1: Fig. S4F, G). And the BS results also showed that DNA methylation level of *H19* in oocytes was low (Additional file 1: Fig. S4H). These data suggest that our samples are not contaminated by somatic cells.

### Altered DMRs methylation in F1 oocytes is transmitted to F2 tissues

**Fig. 6:**
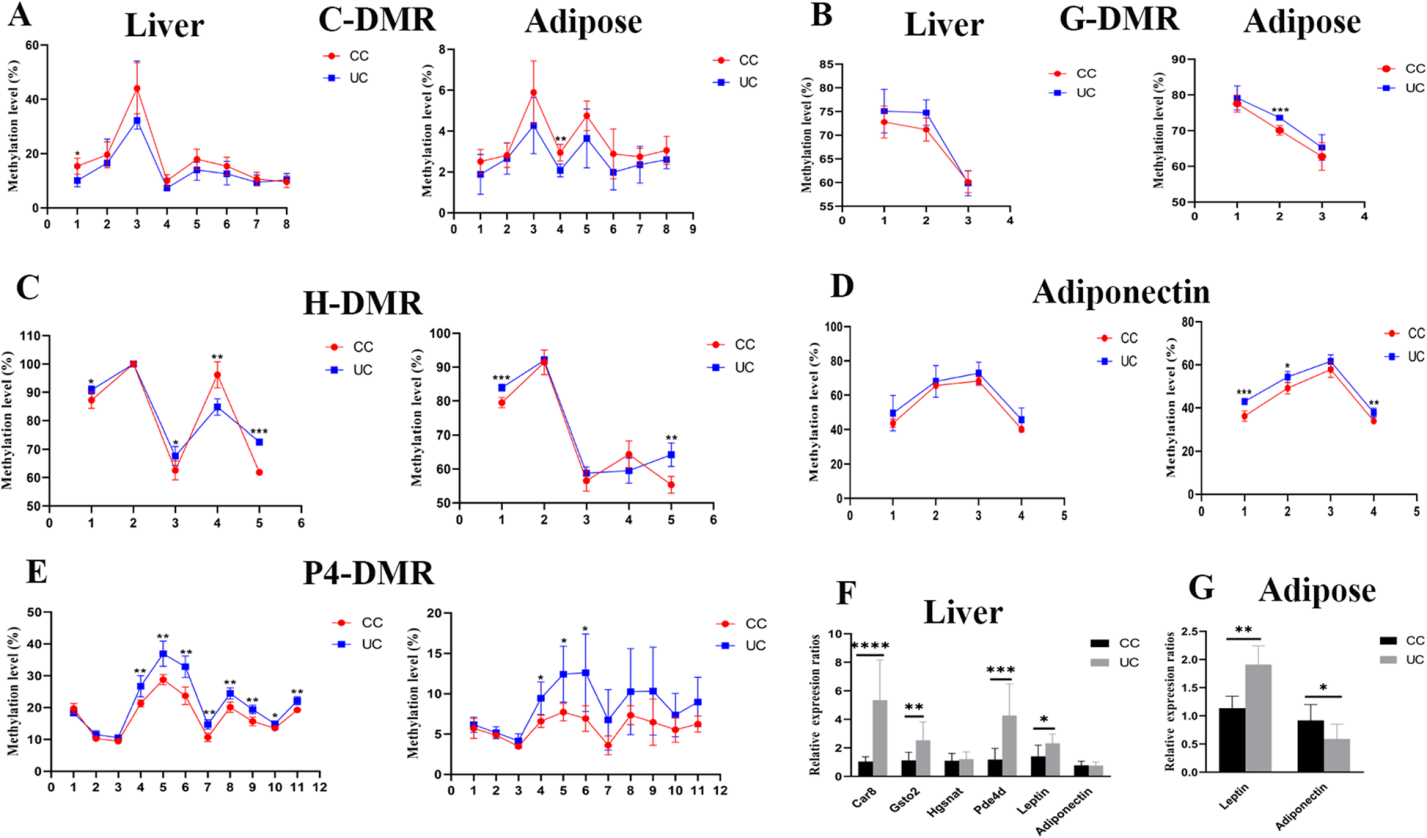
DMRs methylation and mRNA expression in F2 UC female tissues. **A**–**E** DMRs methylation levels in liver and adipose were examined using pyrosequencing, *X*-axial is the CpG sites which were tested. **F** The relative expression of genes related to DMRs was examined using qPCR in liver. **G** The relative expression of *Leptin* and *Adiponectin* in adipose. *p < 0.05, **p < 0.01, ***p < 0.001, ****p < 0.0001. Data are showed as mean ± SD

After fertilization, there is another round of reprogramming, and the tissue-specific methylation patterns are established during development. To assess whether the methylation changes in UN oocytes can be maintained in F2, we examined DNA methylation levels of some DMRs in UC tissues using pyrosequencing. Compared with the CC group, the methylation level of C-DMR was low in UC livers, but there was no statistical difference besides CpG site 1 (Fig. 6A). The methylation level of G-DMR in UC livers was similar to that in CC (Fig. 6B). The methylation levels of hyper-DMRs, H-DMR, and P4-DMR were significantly higher in UC livers compared with CC (Fig. 6C, D). The methylation level of *Adiponectin* in UC livers was not significantly different compared with CC (Fig. 6E). However, it was significantly higher in UC adipose tissues compared with CC (Fig. 6E). The methylation of *Leptin* in livers and adipose was examined using BS, and it was similar between CC and UC (Additional file 1: Fig. S5C). These results suggest that methylation changes in UN oocytes are partly maintained in UC tissues. The published data sets indicate that the validated DMRs-related genes are involved in metabolism. For example, *Pde4d* inactivates cAMP to inhibit insulin secretion [21], and *Pde4d* expression is regulated by *Leptin* [22]. *Car8* decreases insulin level via reducing *Glp-1* level [23]. *Adiponectin* can reduce the sensitivity of insulin [16]. Therefore, we further examined the expression of these genes in tissues and found that the mRNA expressions of *Car8*, *Gsto2*, and *Pde4d* were significantly higher in UC livers than that in CC (*n* = 10 from 6 litters, Fig. 6F). It is unlikely that the changed expressions of *Gsto2* and *Pde4d* are directly mediated by methylation because the methylation of G-DMR and P4-DMR was higher in UC livers (Fig. 6C and E). But the increased expression of *Car8* may be mediated by the decreased methylation in livers. The altered expression of *Adiponectin* and *Hgsnat* were not observed in UC livers (Fig. 6F). In adipose, the expression of *Car8*, *Hgsnat*, *Pde4d*, and *Gsto2* was not observed (Additional file 1: Fig. S6), but the expression of *Adiponectin* was significantly reduced in UC (Fig. 6G) which may be mediated by the higher methylation in the promoter (Fig. 6E). The significantly increased expression of *Leptin* in livers and adipose (Fig. 6F and G) did not coincide with its methylation level at promoter, and this suggests that more factors are involved in regulating *Leptin* expression [18], such as the altered expression of *Pde4d* in livers. These results suggest that the intergenerational perturbed metabolism induced by undernourishment in utero is, at least partly mediated by methylation changes in UN oocytes.

### The altered DNA methylation of validated DMRs is observed in F2 oocytes

**Fig. 7:**
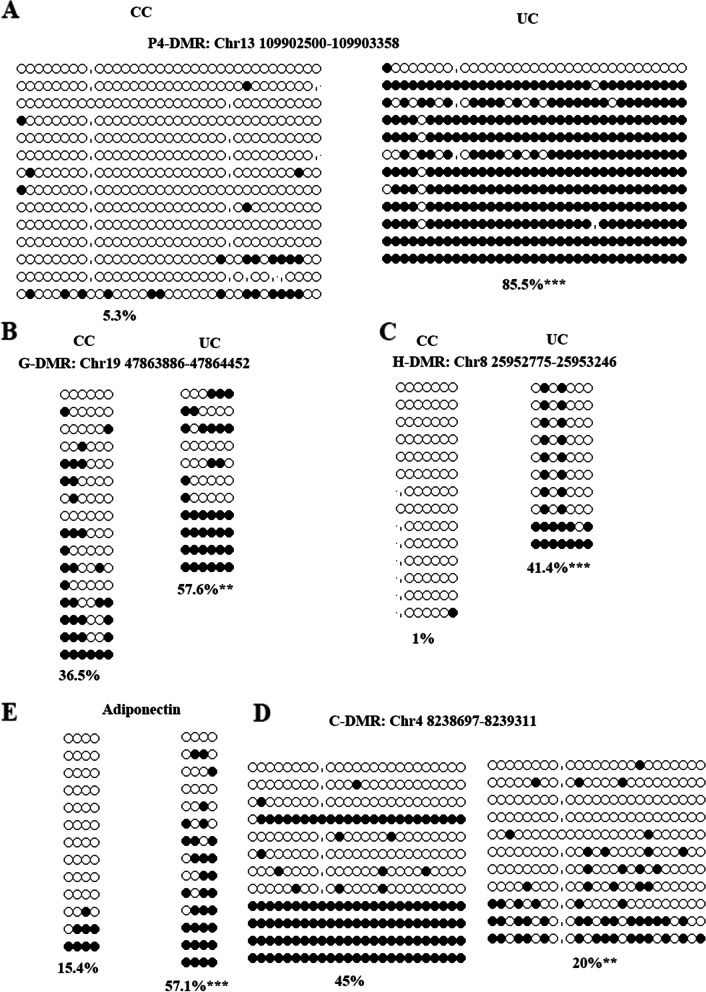
Analysis of DMRs methylation in F2 UC oocytes. To assess the transgenerational inheritance of methylation changes, we examined the DMRs methylation levels in UC oocytes using BS. **A** P4-DMR. **B** G-DMR. **C** H-DMR. **D** C-DMR. **E** Adiponectin. **p < 0.01, ***p < 0.001. Black circle: methylated CG site; white circle: unmethylated CG site

During embryo development, the genome undergoes a reprogramming process including DNA demethylation and remethylation, and tissue-specific methylation patterns are re-established. As shown in Figs. 5 and 6, the methylation patterns of validated DMRs and *Adiponectin* in control oocytes are different (lower) from tissues, and the methylation patterns are tissue-specific. These suggest that the altered methylation of validated DMRs in UN oocytes partially escape the reprogramming during F2 embryo development. So, we wanted to know whether the altered methylation levels of validated DMRs could be, at least partially, maintained in oogenesis. To assess this possibility, we examined the methylation of validated DMRs in UC oocytes using BS. Significant difference of average number of oocytes was not observed between UC and CC groups (Additional file 1: Fig. S7A), but average pups per litter in CC group was significantly higher than that of UC group (Additional file 1: Fig. S7B). Furthermore, we found that the methylation levels of Gn-DMR and P8-DMR in UC oocytes were similar to the CC oocytes (Additional file 1: Fig. S7). But the methylation levels of hyper-DMRs, P4-DMR, G-DMR, and H-DMR were significantly higher in UC oocytes than those in CC oocytes (Fig. 7A, B, and C), and the methylation of C-DMR in UC oocytes was lower than that in CC oocytes (Fig. 7D). Compared with CC, the methylation level of *Adiponectin* was also significantly higher in UC oocytes (Fig. 7E). These results suggest that a part of the altered methylation of validated DMRs in UN oocytes resists the reprogramming during oogenesis, and that may be transmitted to F3.

## Discussion

Metabolic disorders induced by environmental factors can be transmitted to the subsequent generations [24], but the underlying mechanisms are not well known. Our data indicate that the intergenerational transmission of the perturbed metabolism induced by undernourishment in utero may be mediated by DNA methylation via oocytes. We further show that the altered methylation levels of some DMRs in F1 oocytes are maintained in F2 oocytes. This indicates that the methylation changes in oocytes may mediate the transgenerational inheritance of perturbed metabolism induced by malnutrition in utero via oocytes.

Adverse effects of malnutrition in utero on offspring have been widely reported [25]. Starvation during gestation impairs folliculogenesis and germ cell cyst breakdown in offspring [26, 27]. Deficiency of vitamin C during pregnancy alters germ cell meiosis and de-methylation processes [28]. Undernutrition in utero induces abnormal methylation in F1 sperm [14, 20]. Furthermore, the offspring exposed to malnutrition in utero are prone to onset of chronic diseases, such as obesity, diabetes, and cardiovascular disease [29, 30]. Martinez et al. reported that methylation changes in offspring sperm may play a role for the intergenerational transmission of metabolic disorders [14], whereas the altered methylation in offspring sperm is not maintained in the late-gestational somatic tissues of F2 [15]. This indicates that the transgenerational inheritance of disturbed metabolism induced by malnutrition in utero may not be mediated by sperm. A recent study reported that the metabolism of female offspring is more severely affected by maternal malnutrition in utero compared to males [31]. In our present study, we also find that undernutrition in utero disturbs metabolism of F1 females and leads to global hypo-methylation of F1 oocytes. And a part of the altered methylation in F1 oocytes are maintained in adult F2 tissues. Kobayashi et al. show that about half of the DMRs in oocytes are resistant to the global DNA demethylation in preimplantation embryos [31]. These indicate that the abnormal methylation patterns in F1 oocytes induced by the uterine malnutrition may partly escape the reprogramming during early embryo development. And these suggest that the sex dimorphism of effects of malnutrition in utero on offspring may be a reason for the contradiction of the results between females and males [32], and the altered methylation of F1 germ cells could be transmitted to F2 via oocytes.

DNA methylation plays a key role in mediating metabolic disorders, such as diabetes and obesity [33]. We find that a part of methylation changes in F1 oocytes is maintained in F2 tissues. To assess the role of methylation changes in regulating genes expression, we examined the DMRs-related genes expression. Our date showed that the altered expression of several genes in F2 may be regulated by the methylation changes. This disparity is also reported by Radford et al. [14]. These indicate that more factors are involved in mediating the altered gene expression in F2 tissues.

Leptin and adiponectin are two of the most important adipocyte hormones in regulating metabolism. Adiponectin regulates lipid and glucose homeostasis and insulin secretion and function [34]. Leptin is one of the most important factors in regulating glucose metabolism [34] and the function of insulin [34, 35]. Obese mammals have a higher plasma leptin concentration and a lower plasma adiponectin concentration [36]. The imbalance of the circulating leptin and adiponectin levels (leptin/adiponectin ratio, L/A ratio) is an important indicator of metabolic syndromes, such as obesity and diabetes mellitus [37], and it has a long-term effect [38, 39]. In the present study, we find that the expression changes of *Car8* and *Adiponectin* are coincide with the methylation alterations in F2 tissues. Plasma concentration of insulin is decreased which may regulated by the increased expression of *Car8* because higher expression of *Car8* can decrease insulin secretion by negatively regulating Glucagon-like peptide-1 (GLP-1) [23]. And L/A ratio is slightly increased. Recent work confirms that hyper-methylation of *Adiponectin* mediates the metabolic disorders induced by obesity [40], and high L/A ratio means a higher risk of chronic diseases including metabolic diseases [37]. These suggest that methylation changes, at least partly, may mediate the intergenerational transmission of metabolic disorders induced by malnutrition in utero.

There is a reprogramming process in gametogenesis. But ~7% of the global methylation is not erased during oogenesis [41]. If the DMRs methylation changes induced by environmental conditions can escape de-methylation in gametogenesis, it may mediate the transgenerational inheritance of perturbed metabolism. In the present study, we find that the altered methylation of P4-DMR, G-DMR, H-DMR, C-DMR, and *Adiponectin* is maintained in UC oocytes. So, we suggest that the altered methylation of F1 oocytes, at least partly, is transmitted to F2 oocytes which may mediate the transgenerational inheritance of perturbed metabolism. A recent work shows that about half of the DMRs in oocytes are resistant to the global DNA demethylation in preimplantation embryos [31], but how the methylation changes resist reprogramming in oogenesis is unknown. Hill et al. demonstrate that escaping from reprogramming involves a coordinated interplay between PRC1 (polycomb complex), promoter sequence characteristics, DNA methylation, and TET1 [42]. More studies are required to uncover how the methylation changes escape reprogramming during development.

## Conclusions

The present study has provided evidence showing that female germline methylation changes may mediate the transgenerational inheritance of the perturbed metabolism; the DNA methylation changes in F1 oocytes induced by malnutrition in utero may mediate, at least partly, the abnormal expression of metabolism-related genes, causing dysfunction of insulin and metabolic disorders of F2 offspring (Additional file 1: Fig. S8). However, the present study has several limitations. Mouse is an important model animal widely used to investigate human diseases because it is easy to manipulate and its physiology is relatively similar to human compared to lower species [43, 44]. Therefore, in the present study, we used a mouse model to examine the effects of undernutrition in utero on disturbed oocyte methylation and offspring metabolic disorders, whereas we must admit that this cannot completely represent the effects of uterine undernutrition on offspring in human. Therefore, more clinic studies are required for humans. In addition, the molecular mechanisms underlying the inheritance of the altered methylation are still uncovered, especially how some DMRs escape de-methylation remains enigmatic, which needs further clarification. Finally, the metabolism and methylation patterns of F3 are also not investigated.

## Methods

### Study design

It is well known that adverse uterine environment has deleterious influence on offspring health. Studies have demonstrated that uterine malnutrition induces metabolic disorders of F1 offspring and that intergenerational transmission of disturbed metabolism of F1 induced by malnourishment may be mediated by DNA methylation via sperm [20]. Thus, we want to know whether oocyte can mediate the transgenerational inheritance of disturbed metabolism induced by uterine malnutrition via methylation. In the present study, we established a uterine malnutrition mouse model and studied the effects of malnutrition in utero on genomic methylation of F1 oocytes. F2 was produced using female F1 and normal males. And then, we analyzed the DMRs in F1 oocytes and the metabolism of F1 and F2. We further analyzed the methylation and expression of identified genes associated with metabolism in livers and adipose of F1 and F2. The methylation changes of those genes in F2 oocytes were also analyzed.

### Animals

All animal manipulation procedures were approved by the Ethical Committee for Animal Experiments of the Qingdao Agricultural University, China. CD-1 mice were used in our experiments. Mice were housed in an animal facility with 21–23 °C and a 12:12-h light: dark cycle. The mouse model was produced as previously described [20]. To exclude the effect from father, the same males (*n* = 10) was used to produce F1 and F2 offspring. Briefly, estrous female CD-1 mice at 6–8 weeks of age were mated with CD-1 males. Mice with a vaginal plug at the next morning were defined as embryonic day 0.5 (E0.5). Pregnant females were fed with free access to water and standard laboratory chow (Beijing Keao Xieli Feed Co., Ltd., Beijing, China; protein ≥ 200 g/kg, fat ≥ 40 g/kg, fiber ≤ 50 g/kg, lysine 13.2 g/kg, methionine ≥ 7.8 g/kg, vitamin A ≥ 14000 IU/kg, and vitamin D ≥ 1500 IU/kg). On E12.5, females were randomly divided into two groups: control group with free access to chow and undernutrition group with 50% lower nutrients relative to the mean values of the daily consumption during day 0.5 to day 12.5. Food intake of undernutrition mothers was restricted from E12.5 to E18.5, just before the night of parturition. The offspring of control (Con) and undernutrition (UN) dams were fed ad libitum after delivery. The number and body weight of neonatal pups in each litter were recorded within 12 h after birth. Then the litter size was culled to ≤ 8 (randomly selected). At 21days post-partum (21 dpp), Con and UN pups were weaned. The female and male F1 adults were used to obtain F2 offspring as described in Fig. 1A: (i) Con♀-Con♂, CC; and (iii) UN♀-Con♂, UC. At least 12 F1 mice were used to produce F2 for each group. The body weight of pups was recorded every week from birth to 12 weeks of age.

### Glucose (GTT) and insulin tolerance test (ITT)

GTT and ITT were examined as described in a previous study [45]. Briefly, mice at 8 weeks of age were given an intraperitoneal injection of glucose at 2 g/Kg body weight after 16 h fasting. Blood glucose levels were monitored at 0, 30, 60, 90, and 120 min after glucose injection using tail blood. ITT were performed after 4 h fasting. Baseline glucose levels were assessed before the intraperitoneal injection of insulin (Actrapid®, Novo Nordisk). A single intraperitoneal injection of insulin at 10 IU/Kg body weight was delivered. Blood glucose was then measured at 30, 60, 90, and 120 min after insulin injection.

### Oocyte and tissue collection

Metaphase II oocytes were collected from 7 to 8 weeks old females. Mice were superovulated by an intraperitoneal injection of 8 IU pregnant mare serum gonadotropin (PMSG, The SeCond Hormone Factory of Ningbo, Ningbo, China) and 48 h later, followed by an injection of 8 IU human chorionic gonadotropin (hCG, The Second Hormone Factory of Ningbo, Ningbo, China). At 13–14 h of hCG injection, oviductal ampullae were collected into pre-warmed M2 medium. Cumulus-oocyte complexes (COCs) were obtained and then the cumulus cells were removed using 1 mg/ml hyaluronidase. Oocytes were washed with M2 medium until no cumulus cells attached to oocytes. Then, they were collected into 0.2-mL tubes quickly and stored at – 20 °C until used.

The livers, abdominal white adipose tissues, pancreas, and blood were quickly collected when the mice were killed. Tissues and blood were immediately frozen in liquid nitrogen and stored at – 80 °C until used.

### DNA methylation analysis

Tissues were treated using EZ DNA Methylation-Direct Kit (Zymo Research) according to the manufacturer’s instructions. Briefly, 30 mg tissues were used for the bisulfite treatment. Tissues were added to tubes with bisulfite buffer and incubated at 50 °C for 4 h. After that, the treated DNA was purified and stored at − 20 °C for further use.

For oocytes, DNA bisulfite treatment was carried out according to our previous procedures [46]. Briefly, oocytes were lysed using lysis solution (0.04 mg/mL proteinase K, Roche Diagnostics) at 37 °C for 30 min, followed by denaturing in 0.3 M NaOH at 37 °C for 15 min. Then, 15 μL of melted 2% low melting point agarose (Sigma) was added. The mixtures were pipetted into chilled mineral oil and incubated 10 min on ice. The cooled agarose beads were carefully transferred into a fresh 2-mL Eppendorf tube with 500 mL of freshly made bisulfite solution (2.5 M sodium metabisulfite, Merck; 125 mM hydroquinone, Sigma; pH 5) and 200 μL mineral oil, followed by incubation at 50 °C for 4 h. After that, the beads were washed 3 times with 1 mL Tris-EDTA buffer, followed by washing twice with 0.5 mL 0.3 M NaOH and another wash with Tris-EDTA buffer.

The bisulfite-treated DNA was used as templates to amplify the target genes. The DNA fragment was amplified using nested PCR. Primers used in our experiments were presented in Table S1. A total of 80–100 oocytes were used to analyze the DNA methylation level of each gene.

To examine the DNA methylation status of imprinted genes in oocytes, the nested PCR products were digested by restriction endonucleases (COBRA). Two restriction endonucleases were used for each imprinted gene. The PCR products of *H19* were digested with *Taq*^*a*^*I* (recognition site T/CGA) and *RsaI* (GTAC/), and the PCR products of *Igf2r* were digested with *Taq*^*a*^*I* and *BstUI* (CG/CG). The reaction without enzymes was used as negative control. The restriction endonucleases were from New England Biolabs.

To further investigate the DNA methylation level, bisulfite sequencing (BS) was used. The PCR products of all samples for each group were pooled together and cloned into T-vector (Takara) and sequenced (GENEWIZ Company). At least 10 available clones were used for each gene. The methylation levels were analyzed using BiQ Analyzer [47].

### ELISA

We measured the plasma concentration of leptin (Sangon Bioengineering, Shanghai), insulin (Institute of Bioengineering Institute, Nanjing), and adiponectin (Institute of Bioengineering Institute, Nanjing) using ELISA kits according to the manufacturer’s instructions. Briefly, a dilution series of the positive control was made to produce a standard curve by running in triplicate. Samples were diluted into a proper final concentration. Each sample was also run in triplicate. Fifty microliters of standard, sample, or blank was added to each well, covered with a plate sealer and incubated at room temperature for 2.5 h. Then, it was washed in washing buffer for 4 times. One hundred microliters of detecting antibody working solution was added to wells and incubated for 1 h with gentle shaking. After washing, 100 μl of HRP-streptavidin solution was added and incubated for 45 min with gentle shaking. One hundred microliters of TMB substrate was added after washing and incubated for 30 min. Fifty microliters of stop solution was added, and the OD value was measured using a mCD-1oplate reader at 450 nm immediately. We generated a four-parameter logistic standard curve, and the plasma concentration of hormones was calculated.

### RT-qPCR

The relative gene expression was analyzed using RT-qPCR. Briefly, total RNA was extracted using RNAprep pure Tissue Kit (Tiangen, China) according to the manual procedure. The first-strand cDNA was synthesized using Quantscript RT Kit (Tiangen, China). cDNA was used as the template for qPCR. Each sample was run in triplicate. *Gapdh* and *Ppia* were used as control, and the relative expression was calculated using 2^−ΔΔCt^. Primers were presented in Additional file 1: Table S3.

### Global genomic methylation sequencing

We used single-cell methylation sequencing method to examine the methylome in oocytes [48]. A total of 100 MII oocytes were pooled for each sample. All samples were lysed, and bisulfite conversion was performed using the Imprint DNA Modification Kit (Sigma). The modified samples were used for sequencing library. The quality of sequencing library was tested using Qubit 2.0, Agilent 2100, and qPCR. Sequencing was performed using Illumina HiSeq/NovaSeq. The sequenced raw data was evaluated using FastQC, and low-quality data was filtered using trim. The clean data was mapped to reference genome (GRC m38/mm10) using Bismark. Differentially methylated regions were analyzed using DSS.

### Pyrosequencing

DNA of tissues was modified using EZ DNA Methylation Kit (Zymo Research). Methylation level of local-specific regions was analyzed using pyrosequencing. Primers (Table S1) were designed using PyroMark Assay Design 2.0. Pyrosequencing was performed with PyroMark Q48 (Qiagen), and results were analyzed by PyroMark CpG software.

### Statistical analysis

Average data, such as body weight, litter size, concentration of hormone, and glucose level, were presented as mean ± SD (standard deviation), and the statistical significance was analyzed using Student’s *t*-test. Methylation level was presented as a percentage, and the statistical significance was tested using the chi-square test. If *p* < 0.05, it was statistically significant.

## Supplementary Information

**Additional file 1: Fig. S1.** Metabolism for male UN. **Fig. S2.** Metabolism for male UC. **Fig. S3.** Global methylation patterns in UN oocytes. **Fig. S4.** Hypo-DMRs methylation in UN oocytes. **Fig. S5.** Methylation changes in UN oocytes and F2 tissues. **Fig. S6.** The expression of genes in UC female adipose. **Fig. S7.** Hypo-DMRs methylation in UC oocytes. **Fig. S8.** Summary of the transgenerational inheritance of the metabolic disorders induced by undernourishment in utero. **Table S1.** Identified DMRs located at promoters. **Table S2.** Enrichment of genes with DMRs in promoters. **Table S3.** Primers used for nested PCR, pyrosequencing, and qPCR.

## Data Availability

The raw data of genomic DNA methylation in oocytes are uploaded at BIG Hub, No. PRJCA005552 (https://ngdc.cncb.ac.cn/gsub/) [49].

## Abbreviations

DMR: Differentially methylated region
UN: Undernourishment in utero
GTT: Glucose tolerance test
ITT: Insulin tolerance test
L/A ratio: Leptin/adiponectin ratio
CC: Offspring of control female with control male
UC: Offspring of UN female with control male
MII: Metaphase II
mC: Methylated cytosine
CG: Cytosine-guanine nucleotide
UTR5: 5′ Untranslated region
TSS: Transcriptional start site
TES: Transcriptional end site
Hyper-DMRs: Hyper-methylated DMRs
Hypo-DMRs: Hypo-methylated DMRs
CGI: CG island
UTR3: 3’ Untranslated region
KEGG: Kyoto Encyclopedia of Genes and Genomes
acs: Acetoacetyl-CoA synthetase
Acsl6: Long-chain acyl-CoA synthetase 6
Adcy8: Adenylate cyclase
Bcat2: Branched-chain aminotransferase 2
Coq5: Coenzyme Q5
Got1/2: Aspartate aminotransferase 1/2
Isyna1: Inositol-3-Phosphate Synthase 1
Pde4d: cAMP phosphodiesterase 4d
Pde10a: cAMP phosphodiesterase 10a
Pde8b: cAMP phosphodiesterase 8b
Plpp2: Phospholipid phosphatase 2
Shmt2: Serine hydroxymethyltransferase 2
Tkt: Transketolase
car8: Carbonic anhydrase 8
Pigl: Phosphatidylinositol glycan anchor biosynthesis class L
H-DMR: DMR at the promoter of Hgsnat
G-DMR: DMR at the promoter of Gsto2
C-DMR: DMR at the promoter of Car8
P4-DMR: DMR at the promoter of Ped4d
Gn-DMR: DMR at the promoter of Gnas
P8-DMR: DMR at the promoter of Pde8b
A-DMR: DMR at the promoter of Acsl6
Lxra: Liver X receptor alpha
CpG: Cytosine-phosphate-guanine
BS: Bisulfite sequencing
H19: H19 imprinted maternally expressed transcript
Igf2r: Insulin like growth factor 2 receptor
Glp1: Glucagon-like peptide-1
PRC1: Polycomb repressive complex 1
TET1: Ten-eleven translocation methylcytosine dioxygenase 1
