# Supplementary material for: Inheritance of perturbed methylation and metabolism caused by uterine malnutrition via oocytes
Source: BMC Biol. 2023 Feb 24;21:43. doi: 10.1186/s12915-023-01545-x (PMC9960220; doi:10.1186/s12915-023-01545-x)
Supplement: Supplementary file 1 — Additional file 1: Fig. S1. Metabolism for male UN. Fig. S2. Metabolism for male UC. Fig. S3. Global methylation patterns in UN oocytes. Fig. S4. Hypo-DMRs methylation in UN oocytes. Fig. S5. Methylation changes in UN oocytes and F2 tissues. Fig. S6. The expression of genes in UC female adipose. Fig. S7. Hypo-DMRs methylation in UC oocytes. Fig. S8. Summary of the transgenerational inheritance of the metabolic disorders induced by undernourishment in utero. Table S1. Identified DMRs located at promoters. Table S2. Enrichment of genes with DMRs in promoters. Table S3. Primers used for nested PCR, pyrosequencing, and qPCR. [file 12915_2023_1545_MOESM1_ESM.pdf]

Additional file 1:

Inheritance of perturbed methylation and metabolism caused by uterine malnutrition via oocytes

Shou-Bin Tang [1#], Ting-Ting Zhang [1, 2#], Shen Yin [1], Wei Shen [1], Shi-Ming Luo[3], Yong Zhao[4], Cui-Lian Zhang [2], Francesca Gioia Klinger[5], Qing-Yuan Sun [3*], Zhao-Jia Ge [1*]

1. College of Life Sciences, Institute of Reproductive Sciences, Key Laboratory of Animal Reproduction and Germplasm Enhancement in Universities of Shandong, Qingdao Agricultural University, Qingdao 266109, P.R. China;

2. Reproductive Medicine Center, People's Hospital of Zhengzhou University, Henan Provincial People's Hospital, Zhengzhou 450003, P.R. China;

3. Fertility Preservation Lab and Guangdong-Hong Kong Metabolism & Reproduction Joint Laboratory, Reproductive Medicine Center, Guangdong Second Provincial General Hospital, Guangzhou 510317, China

4. State Key Laboratory of Animal Nutrition, Institute of Animal Sciences, Chinese Academy of Agricultural Sciences, Beijing, China

5. Histology and Embryology, Saint Camillus International University of Health Sciences, Rome, Italy

# These authors have equal Contribution.

*Corresponding authors: GZJ: +86 15192662096; gejdssd313@163.com; zjge@qau.edu.cn; SQY: sunqy@gd2h.org.cn

Figure legends

Fig. S1 Metabolism for male UN

(A-D), Significantly higher blood glucose level was observed for male and female UN at 8 weeks of age, but not at 12 weeks of age; **(E and F),** The GTT and ITT were examined in male UN, and significantly statistical difference was observed; **(G),** the L/A ratio was calculated, and it was significantly increased in male UN; **(H-J),** the plasma concentrations of leptin, adiponectin, and insulin were examined, and slightly changes were observed;. *P<0.05, **P<0.01.

Fig. S2 Metabolism for male UC and CU

(A and B), GTT and ITT were examined for male UC, and there was no statistical difference between CC and UC; **(C),** the body weights of male UC and CC were also tested at different ages, and it was similar between groups; **(D-F),** the plasma concentrations of leptin, adiponectin, and insulin in male F2 were examined, and there was no statistical difference between groups; but **(G)** the L/A ratio was significantly increased in male UC. *P<0.05.

Fig. S3 Global methylation patterns in UN oocytes

(A), According to the context, C is divided into three subclasses CG, CHG, and CHH (H=A, T, or C). The proportions of different subclasses in control and UN oocytes were shown, and the proportion of CG was significantly decreased in UN oocytes; **(B),** the distribution of global methylation at different regions in oocytes; **(C),** the global methylation was calculated at different regions of genes, including upstream 2K of genes, gene bodies, and downstream 2k of genes.

Fig. S4 Hypo-DMRs methylation in UN oocytes

(A-C), The selected hypo-DMRs methylation in UN oocytes was examined using BS, and at least 10 available clones were showed. The statistical difference of methylation percentage was tested by Chi-square test; **(D),** the methylation level in promoter of *Lxra* in UN oocytes was also examined using BS, and there was no statistical difference; **(E), t**he methylation of leptin in UN oocytes; (F-H), the methylation of imprinted genes *H19* and *Igf2r* was analyzed by COBRA and BS. *P<0.05.

Fig. S5 Methylation changes in UN oocytes and F2 tissues

(A and B), The methylation of *Leptin* and *Adiponectin* in the genomic BS-seq data was examined using IGV software, and the methylation level was higher in UN oocytes at the covered CpG sites; **(C),** the methylation of Leptin in livers and adipose was examined using BS, and it was similar between CC and UC.

Fig. S6 The expression of genes in UC female adipose

The expressions of *Car8*, *Gsto2*, *Hgsnat*, and *Pde4d* in female adipose were examined by qPCR, and *Gapdh* and *Ppia* were used as controls. The result showed that there was no expression of these genes in adipose.

Fig. S7 Hypo-DMRs methylation in UC oocytes

The averages of oocyte numbers and pups per litter for female F2 were calculated, and showed in **(A)** and **(B),** respectively; **(C and D),** the methylation levels of hypo-DMRs in UC were examined using BS.

Fig. S8 Summary of the transgenerational inheritance of the metabolic disorders induced by undernourishment in utero

Tables

Table S1 Identified DMRs located at promoters

Table S2 Enrichment of genes with DMRs in promoters

Table S3 Primers used for nested PCR, pyrosequencing, and qPCR

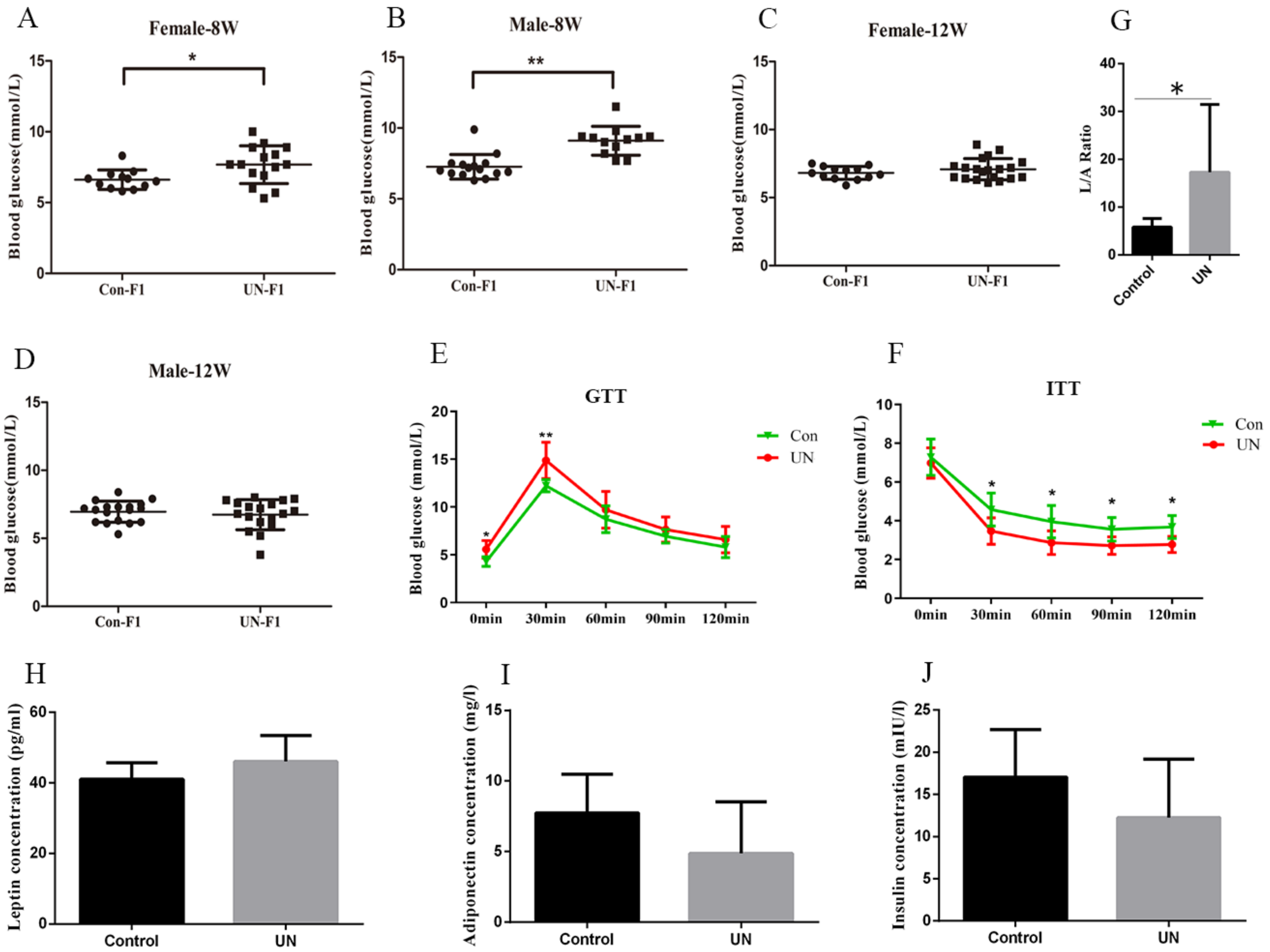
A
Female-8W
Blood glucose(mmol/L)
Con-F1
UN-F1
B
Male-8W
C
Female-12W
G
L/A Ratio
Control
UN
D
Male-12W
E
GTT
Blood glucose (mmol/L)
0min
30min
60min
90min
120min
Con
UN
F
ITT
H
Leptin concentration (pg/ml)
I
Adiponectin concentration (mg/l)
J
Insulin concentration (mIU/l)

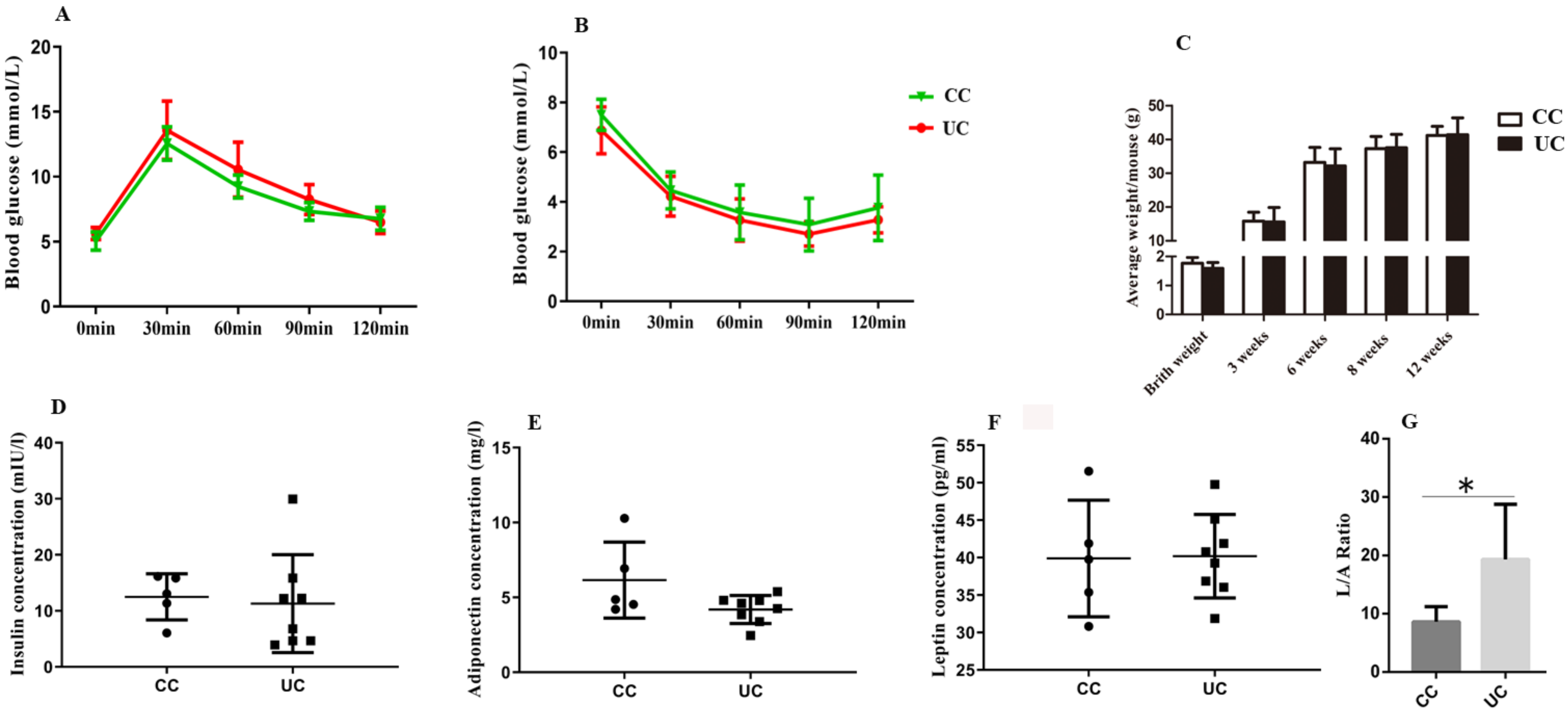

A
Blood glucose (mmol/L)
0min
30min
60min
90min
120min
B
Blood glucose (mmol/L)
CC
UC
C
Average weight/mouse (g)
Brith weight
3 weeks
6 weeks
8 weeks
12 weeks
D
Insulin concentration (mIU/l)
E
Adiponectin concentration (mg/l)
F
Leptin concentration (pg/ml)
G
L/A Ratio
*

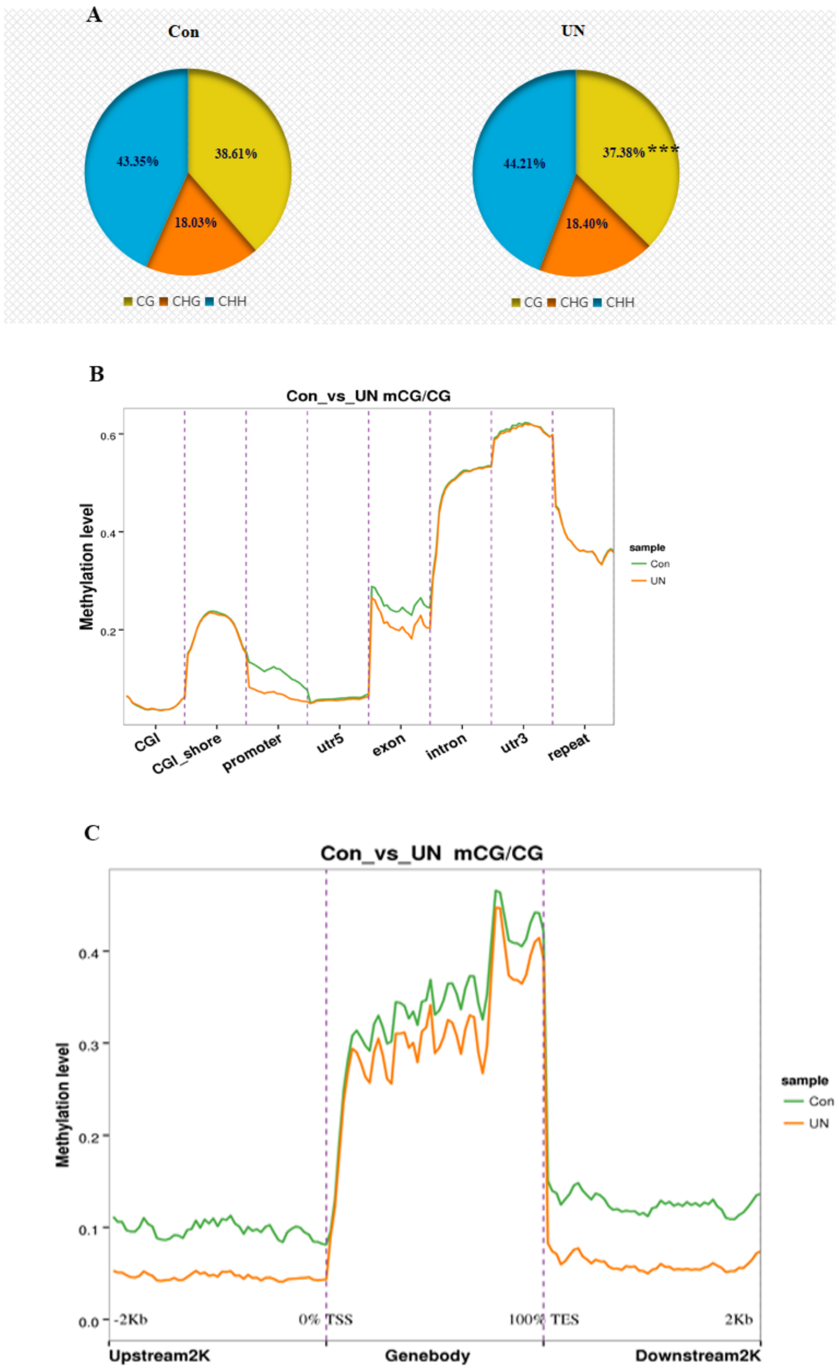
A
Con
43.35%
38.61%
18.03%
CG CHG CHH
UN
44.21%
37.38%***
18.40%
CG CHG CHH
B
Con_vs_UN mCG/CG
Methylation level
0.6
0.4
0.2
CGI
CGI_shore
promoter
utr5
exon
intron
utr3
repeat
sample
Con
UN
C
Con_vs_UN mCG/CG
Methylation level
0.4
0.3
0.2
0.1
0.0
-2Kb
0% TSS
100% TES
2Kb
Upstream2K
Genebody
Downstream2K
sample
Con
UN

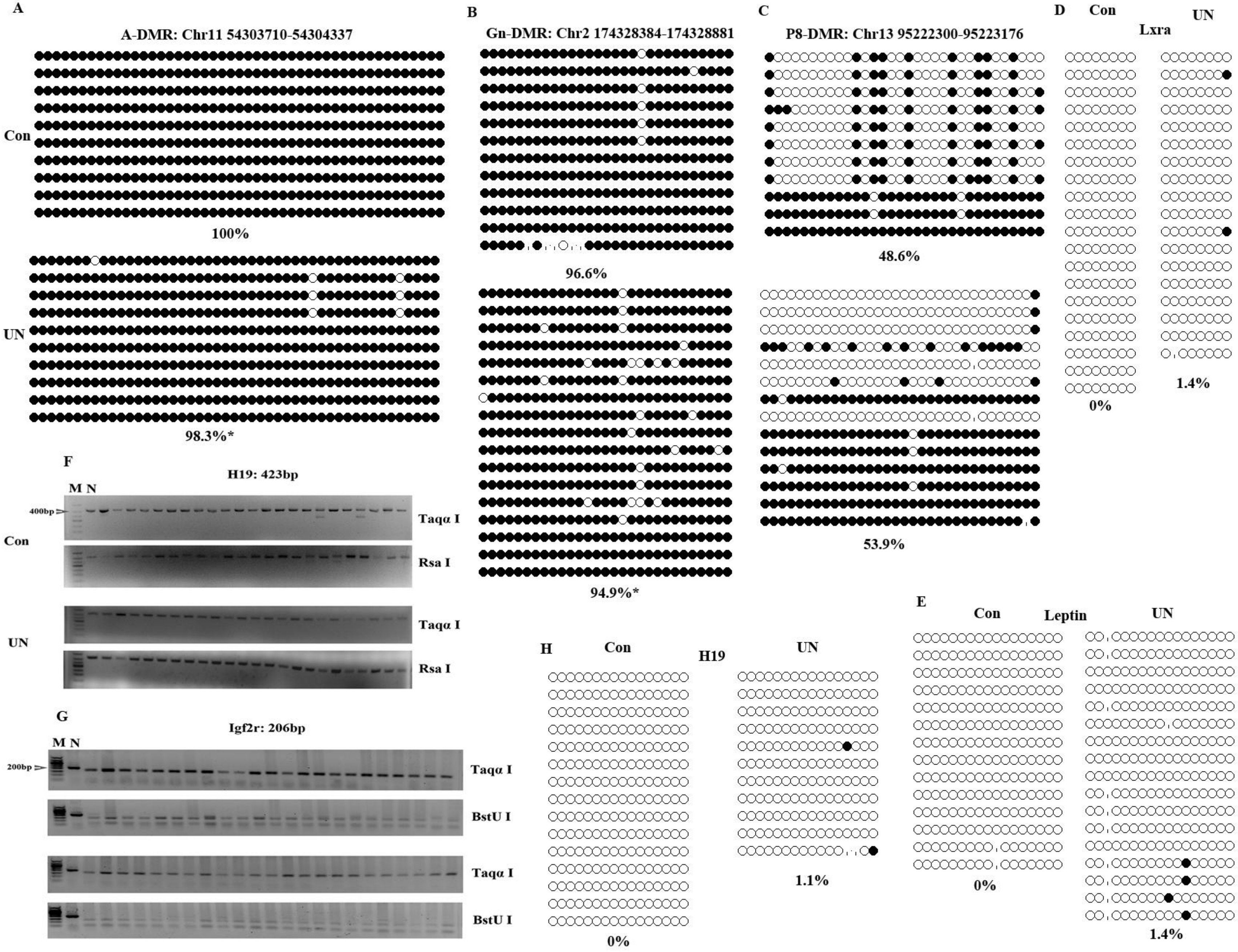
A
A-DMR: Chr11 54303710-54304337
Con
100%
UN
98.3%*
B
Gn-DMR: Chr2 174328384-174328881
96.6%
94.9%*
C
P8-DMR: Chr13 95222300-95223176
48.6%
53.9%
D
Con
Lxra
UN
0%
1.4%
E
Con
Leptin
UN
0%
1.4%
F
H19: 423bp
M N
400bp
Con
Taqα I
Rsa I
UN
Taqα I
Rsa I
G
Igf2r: 206bp
M N
200bp
Taqα I
BstU I
Taqα I
BstU I
H
Con
H19
UN
0%
1.1%

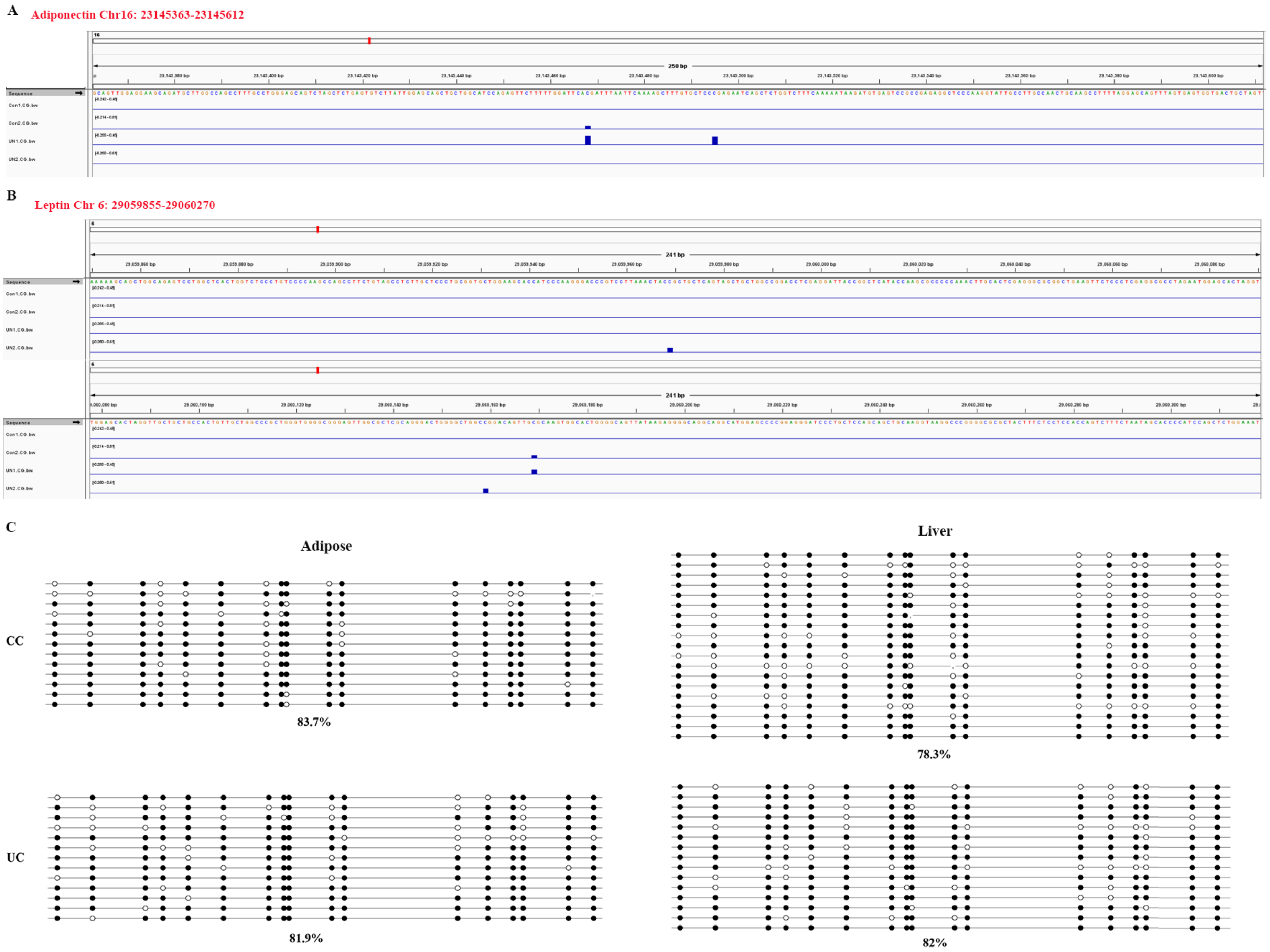
A
Adiponectin Chr16: 23145363-23145612
250 bp
Sequence
Con1.CG.bw
Con2.CG.bw
UN1.CG.bw
UN2.CG.bw
B
Leptin Chr 6: 29059855-29060270
241 bp
C
Adipose
Liver
CC
UC
83.7%
78.3%
81.9%
82%

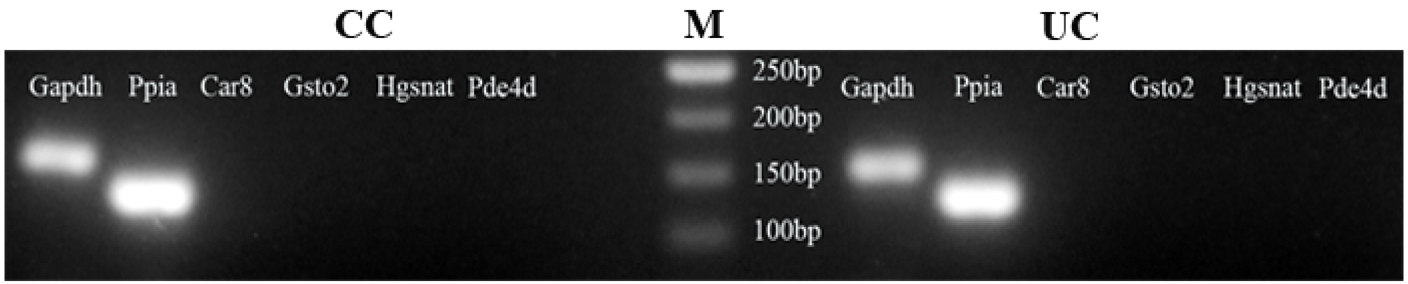
CC
M
UC
Gapdh
Ppia
Car8
Gsto2
Hgsnat
Pde4d
250bp
200bp
150bp
100bp
Gapdh
Ppia
Car8
Gsto2
Hgsnat
Pde4d

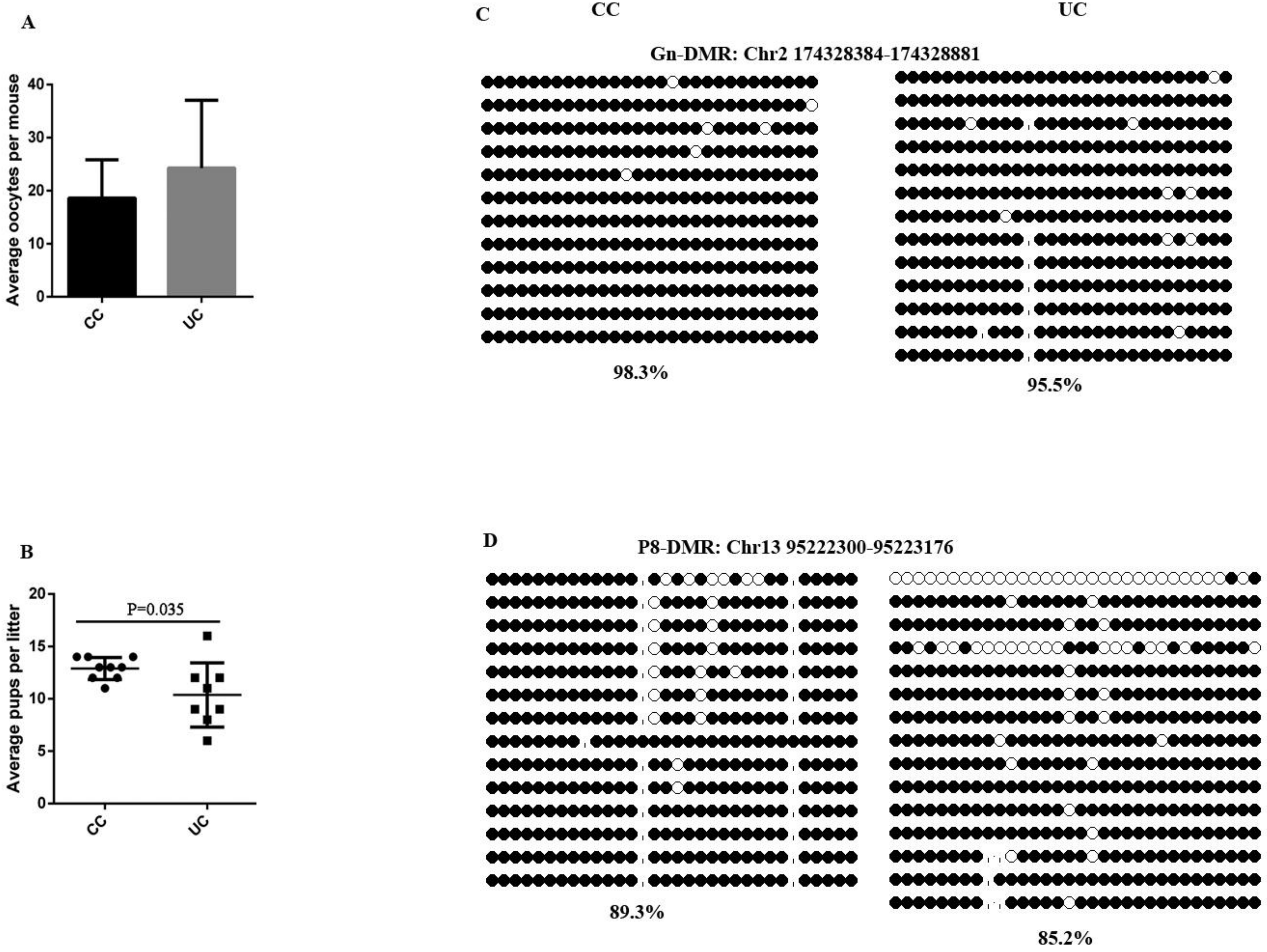
A
Average oocytes per mouse
40
30
20
10
0
CC
UC
B
Average pups per litter
20
15
10
5
0
P=0.035
CC
UC
C
CC
UC
Gn-DMR: Chr2 174328384-174328881
98.3%
95.5%
D
P8-DMR: Chr13 95222300-95223176
89.3%
85.2%

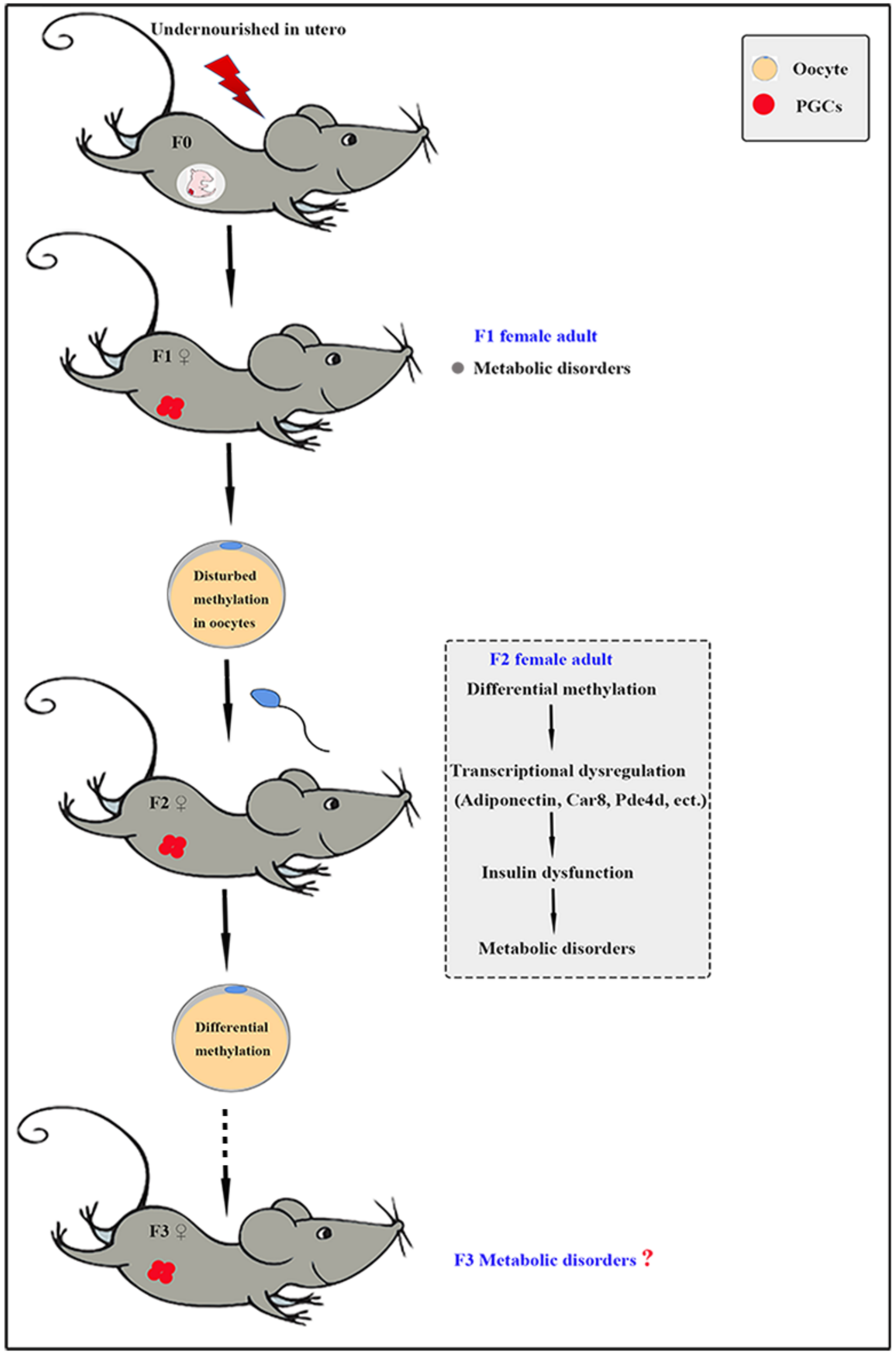

Undernourished in utero
Oocyte
PGCs
F0
F1 ♀
F1 female adult
Metabolic disorders
Disturbed methylation in oocytes
F2 female adult
Differential methylation
Transcriptional dysregulation
(Adiponectin, Car8, Pde4d, ect.)
Insulin dysfunction
Metabolic disorders
F2 ♀
Differential methylation
F3 ♀
F3 Metabolic disorders ?
